# Graphene Oxide-Regulated Calcium Alginate Molecularly Imprinted Hydrogel Films with Enhanced Physico-Mechanical Properties and Selective Protein Recognition

**DOI:** 10.3390/gels12100913

**Published:** 2026-10-10

**Authors:** Siyu Wang, Haojie Li, Ying Zhang, Zhengchun Yang, Kongyin Zhao

**Affiliations:** 1 State Key Laboratory of Advanced Separation Membrane Materials, School of Material Science and Engineering, Tiangong University, Tianjin 300387, China; 2431020315@tiangong.edu.cn (S.W.); 2531020359@tiangong.edu.cn (H.L.); 2530020266@tiangong.edu.cn (Y.Z.); 2 School of Integrated Circuit Science and Engineering, Advanced Materials and Printed Electronics Center, Tianjin University of Technology, Tianjin 300384, China

**Keywords:** graphene oxide, calcium alginate hydrogel, molecularly imprinted polymer, protein recognition, electrode interface, antibacterial activity

## Abstract

Calcium alginate (CaAlg) hydrogels are attractive matrices for molecular imprinting because of their biocompatibility and functionalizability, but their limited mechanical stability and excessive swelling can compromise the integrity and accessibility of molecular recognition sites. In this study, graphene oxide (GO) was incorporated into CaAlg to regulate the physicochemical properties of the hydrogel and construct a bovine serum albumin (BSA)-imprinted hydrogel interface for selective protein recognition and electrochemical sensing. The incorporation of GO effectively reduced swelling and enhanced the mechanical stability of the hydrogel, with the 1 wt.% GO-containing film exhibiting a tensile strength of 1921.15 kPa and the highest fracture energy. The resulting molecularly imprinted hydrogel showed a BSA adsorption capacity of 55.21 mg g^−1^ and an imprinting efficiency of 3.68, while preferential recognition of BSA over other proteins was observed. After integration with a carbon electrode, template removal and BSA rebinding produced distinguishable electrochemical responses, with an electrochemical imprinting ratio of approximately 6.5. These results demonstrate that GO can simultaneously regulate the structural stability and molecular recognition behavior of CaAlg-based imprinted hydrogels. The proposed hydrogel interface provides a promising material platform for selective protein recognition and electrochemical sensing, with potential applications in biomolecular detection and functional biointerfaces.

## 1. Introduction

The development of efficient and accurate target molecule identification technologies remains a critical pursuit in modern analytical science [1,2]. Molecular imprinting technology (MIT) addresses this need by creating molecularly imprinted polymer (MIP) with tailored recognition sites [3,4,5]. This process involves polymerizing functional monomers around a template molecule, followed by its removal, resulting in “imprinted cavities” that exhibit high complementarity to the template’s spatial structure and binding sites [6]. Functionally analogous to natural antigen–antibody systems, MIT offers distinct advantages including controllable synthesis, low cost, high stability, and reusability [7,8,9]. These attributes have driven its successful application in solid-phase extraction [10,11,12], chromatographic separation [13,14,15], and chemical sensing [16,17,18].

Molecularly imprinted sensors, integrating MIPs with transduction mechanisms (e.g., electrochemical, optical, and thermometric), constitute an important class of detection platforms [19]. The immobilization of MIPs on electrode surfaces provides molecular recognition capability, thereby improving the selectivity of the resulting interfaces [20,21]. Among them, Ayankojo et al. [22] fabricated erythromycin-imprinted poly(m-phenylenediamine) films via electrochemical polymerization, achieving trace-level erythromycin detection; Lu et al. [23] engineered a dopamine-selective poly(nicotinamide) electrode with rapid rebinding behavior; Kaya et al. [24] developed electrochemical platforms for the determination of endocrine disruptors including bisphenol A and phthalate derivatives. Xu et al. [25] developed molecularly imprinted polymer-coated hybrid optical waveguides for ultrasensitive fluorescence sensing. Nevertheless, conventional MIP systems remain fundamentally constrained by limited recognition-site accessibility and inefficient mass transfer, which may restrict their sensing performance. Concurrently, graphene oxide (GO) has emerged as a highly promising nanomaterial due to its unique two-dimensional structure, large specific surface area, and abundant surface functional groups [26,27,28]. The incorporation of GO into hydrogel networks can improve their mechanical properties and regulate swelling behavior [29,30], providing a potential strategy for enhancing the structural stability and protein-recognition performance of CaAlg-based imprinted hydrogel films. Bovine serum albumin (BSA) is widely used as a model template protein in molecularly imprinted polymer systems because of its relatively low cost and easy availability, and its extensive use provides a well-established basis for evaluating protein recognition performance [31]. Although the relatively large molecular size and conformational flexibility of proteins can pose challenges for molecular imprinting, previous studies have demonstrated that BSA can be successfully employed as a template for constructing selective molecularly imprinted hydrogels [32]. Therefore, BSA was selected as the model template protein to evaluate the ability of hydrogel matrices to establish selective recognition sites for a relatively large protein.

Previous studies have demonstrated that the incorporation of functional fillers into CaAlg-based molecularly imprinted systems can improve their physicochemical and mechanical properties. However, integrating mechanical reinforcement with selective molecular recognition, electrochemical sensing, and additional antibacterial functionality within a single CaAlg-based imprinted hydrogel remains challenging. In this study, we developed a GO-enhanced calcium alginate (CaAlg) hydrogel interface (GO/CaAlg) for molecularly imprinted electrochemical sensing. GO-doped sodium alginate (NaAlg) was employed as the functional hydrogel matrix, bovine serum albumin (BSA) was selected as the template protein, and CaCl_2_ was used as the ionic crosslinker to fabricate BSA-imprinted MIP hydrogel films and corresponding non-imprinted polymer (NIP) controls. Different from conventional CaAlg-based imprinted systems that mainly suffer from insufficient mechanical stability, excessive swelling, and limited interfacial charge transfer, the present design emphasizes the regulatory role of GO at the hydrogel/electrode interface. The incorporation of GO not only reinforces the CaAlg network and improves its anti-swelling capability, but also contributes to the formation of more accessible imprinted cavities and facilitates charge transport across the modified electrode interface. The resulting material was systematically characterized in terms of physicochemical properties, mechanical performance, swelling behavior, BSA adsorption and imprinting characteristics, electrochemical sensing response, and antibacterial activity.

## 2. Results and Discussion

### 2.1. Characterization of Protein Imprinted GO/CaAlg Hydrogel Film

**Figure 1:**
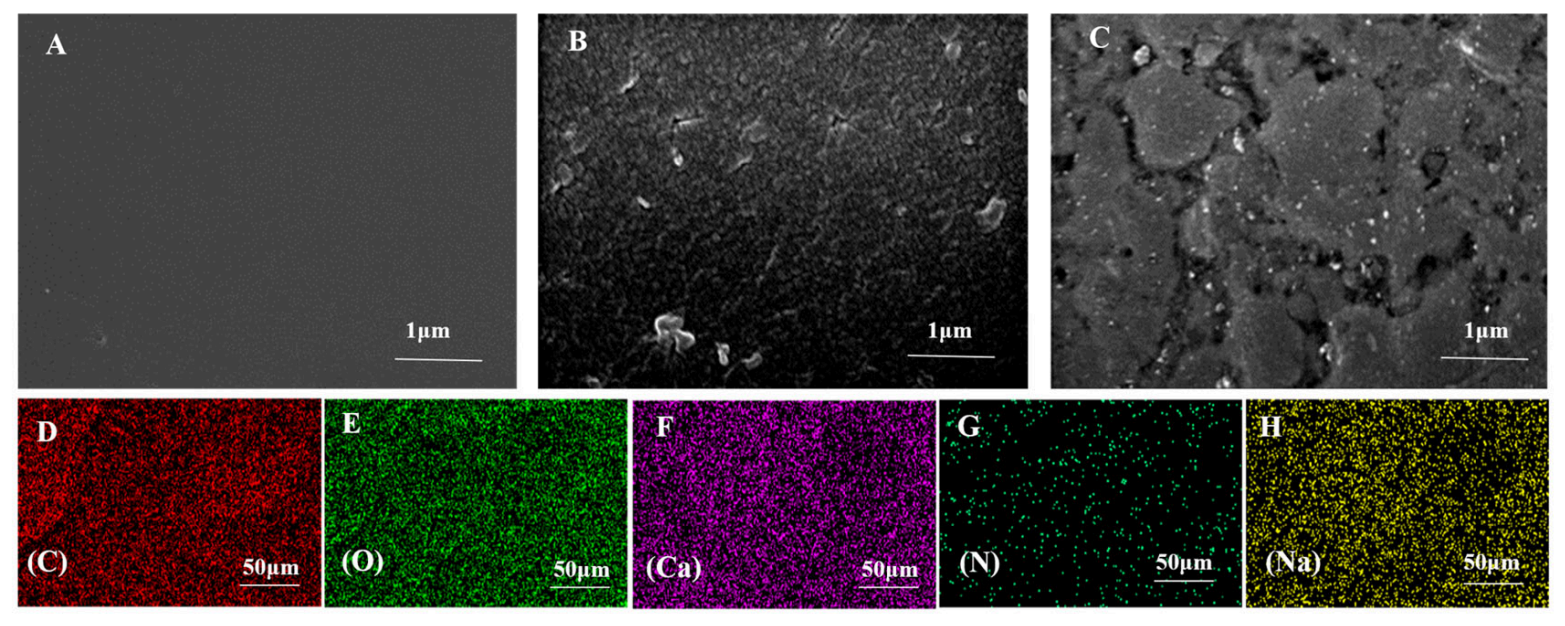
SEM images of the pristine CaAlg film (**A**) and GO/CaAlg MIP films before (**B**) and after removing BSA (**C**) and EDS elemental mapping of the GO/CaAlg MIP film after removing BSA (**D**–**H**).

The surface morphology of the hydrogel films was characterized by SEM. As shown in Figure 1A, the pristine CaAlg film exhibited a relatively smooth and continuous surface without obvious large-scale defects. Similar surface characteristics have been reported for CaAlg films formed through Ca^2+^-mediated ionic cross-linking [33]. Following the incorporation of GO and BSA during MIP formation, a distinct morphological change was observed. Specifically, the MIP film before elution exhibited a rougher surface with irregular features (Figure 1B), which may be associated with the incorporation and interactions of BSA and GO within the CaAlg matrix. After elution of the template BSA using Tris-HCl solution, more pronounced groove-like and heterogeneous structures appeared on the surface of the imprinted film (Figure 1C). Similar morphological changes following template removal have been reported in molecularly imprinted hydrogel systems [34]. These changes may be related to the release of BSA and the accompanying local rearrangement of the hydrogel network.

It should be noted that soft alginate-based hydrogels may undergo partial dehydration and structural shrinkage under the high-vacuum and electron-beam conditions required for SEM imaging, which can affect the preservation of their native hydrated morphology and the direct comparison of surface features across different magnifications. Nevertheless, the SEM images of the pristine CaAlg film (Figure 1A), the GO/BSA-incorporated MIP film before template removal (Figure 1B), and the imprinted film after template removal (Figure 1C) show discernible differences in surface morphology. In particular, the relatively smooth surface of the pristine CaAlg film became rougher after incorporation of GO and BSA, while additional surface features were observed after elution. These morphological changes are consistent with the formation of a modified hydrogel matrix and the subsequent removal of the template protein. Similar morphological characteristics have been reported for GO/CaAlg hydrogel networks, where GO nanosheets were incorporated into the alginate matrix while maintaining the overall structural integrity of the hydrogel film [35].

Figure 1D–H show the EDS elemental mapping of the GO/CaAlg MIP film after removing BSA, illustrating the distribution of five elements—C, O, Ca, N, and Na—within the hydrogel film. All elements are uniformly distributed. Table 1 lists the weight percentage contents of these elements. Among them, C exhibits the highest content at 50.61 wt.%, followed by O at 40.93 wt.%, while Ca, Na, and N account for 4.66, 2.78, and 1.02 wt.%, respectively.

**Figure 2:**
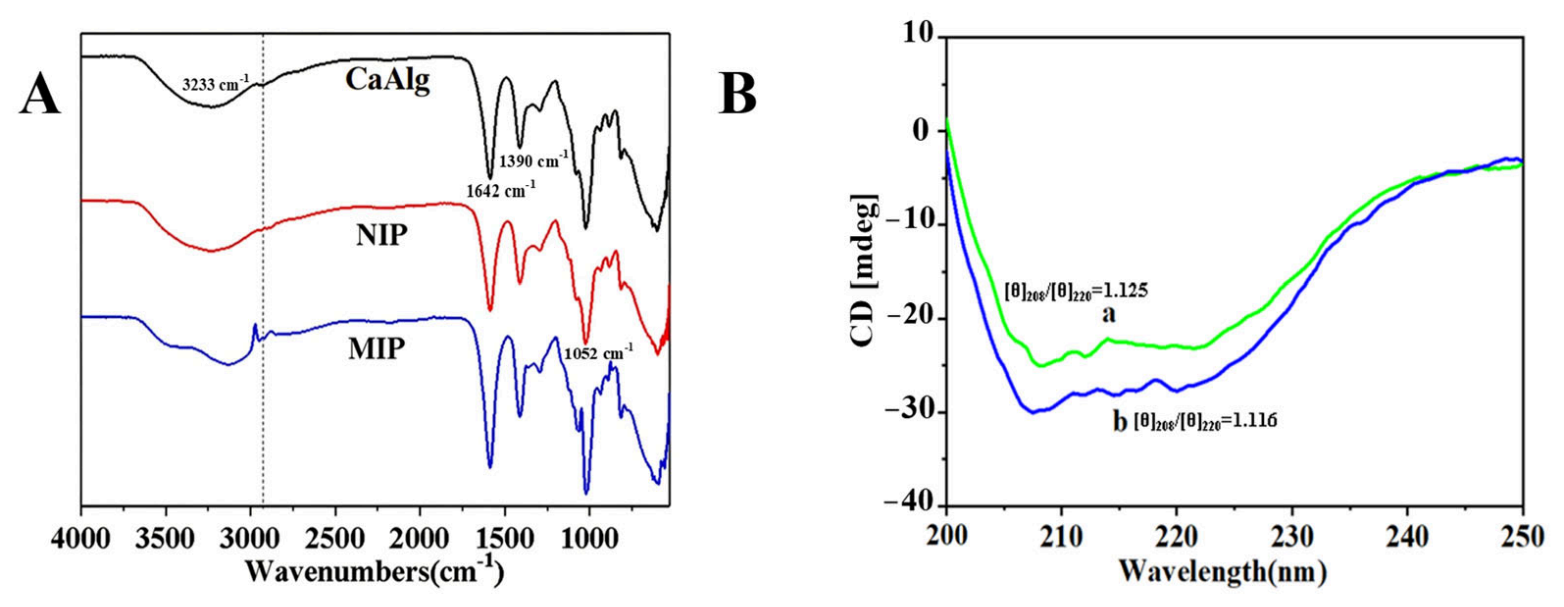
Infrared spectra of CaAlg, MIP, and NIP films (**A**), circular dichroism spectra of BSA solution after elution of MIP film with Tris-HCl solution (a) and original BSA aqueous solution (b) (**B**).

Qualitative analysis of the hydrogel chemical group composition was performed using FTIR spectroscopy. Figure 2A shows the infrared spectra of CaAlg, GO/CaAlg (NIP), and GO/CaAlg (MIP) hydrogel films. In the CaAlg spectrum, characteristic absorption peaks appear at 3233 cm^−1^ (O–H stretching vibration), 1642 cm^−1^ (C=O stretching vibration), and 1390 cm^−1^ (–OH bending vibration) [36]. Similar peaks are observed in the NIP and MIP spectra. Notably, the MIP film exhibits a distinct C-O stretching vibration peak of GO at 1052 cm^−1^, supporting the successful incorporation of oxidized graphene into the MIP network [37].

To evaluate potential structural changes in template protein, the effect of MIP films on protein secondary structure was systematically studied using CD spectroscopy [38]. As shown in Figure 2B, the CD spectrum of the prepared BSA solution reveals characteristic negative ellipticity peaks at 220 nm and 208 nm, consistent with a predominantly α-helical secondary structure [39]. The similar [θ]_208_/[θ]_220_ ratios of 1.116 and 1.125 indicate that no substantial alteration in the secondary structure of BSA occurred during film preparation and template removal [40].

### 2.2. Physico-Mechanical Properties of GO/CaAlg Films

**Figure 3:**
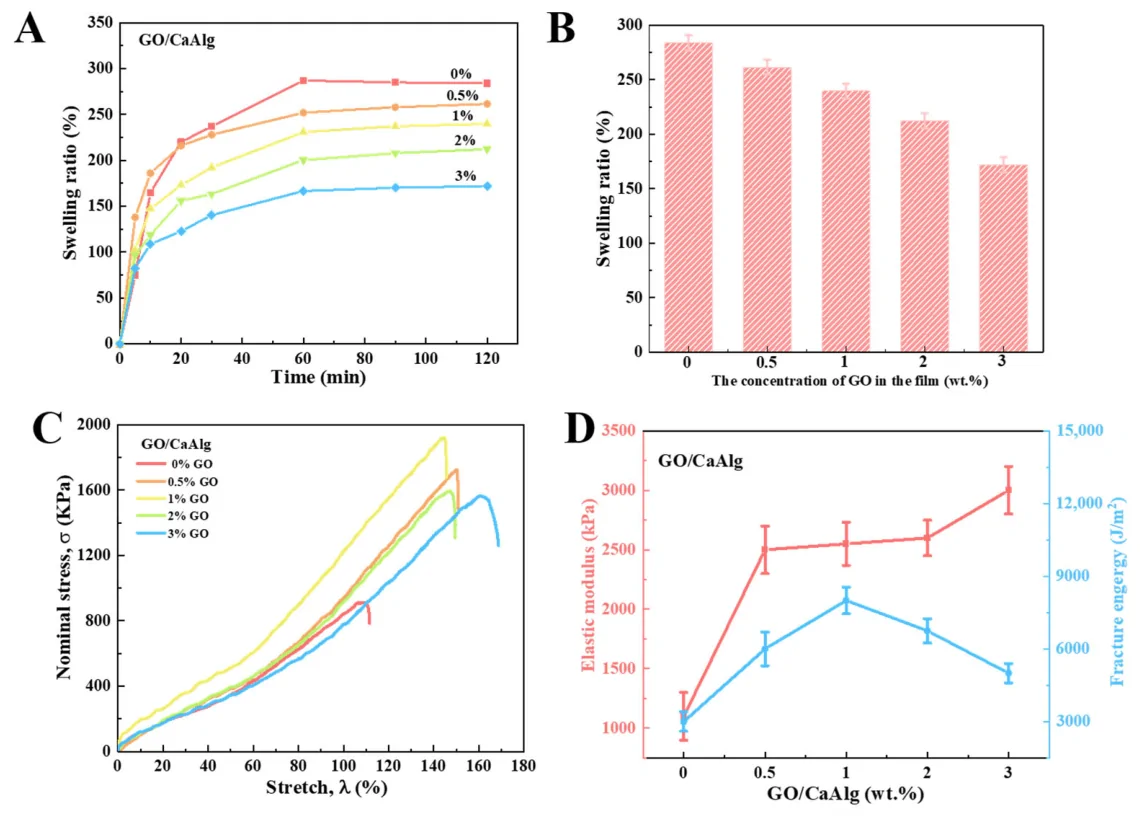
Swelling curves of GO/CaAlg films with different GO contents (**A**), corresponding equilibrium swelling ratios (**B**), stress–strain curves (**C**), elastic modulus and fracture energy (**D**).

Following morphological and chemical characterization, the physico-mechanical properties (swelling resistance and mechanical strength) of the hydrogels were investigated. Figure 3A presents the swelling kinetics and equilibrium swelling ratios of GO/CaAlg films with varying GO loadings. All samples exhibited an initial increase in swelling rate followed by a gradual plateau. Similar swelling behavior has been reported for CaAlg-based hydrogels and has been associated with the gradual loss of Ca^2+^, which weakens the ionic cross-linked network during immersion [41]. Therefore, the observed swelling behavior may be related to changes in the ionic cross-linked structure of the CaAlg network during immersion. Critically, increasing GO loading significantly reduced swelling rates (Figure 3A). The decrease in swelling ratio may be associated with additional interactions between GO and the CaAlg network, which restrict polymer-chain mobility and network expansion [42,43]. To benchmark the swelling behavior, the equilibrium swelling ratio of the GO/CaAlg film containing 1 wt.% GO (Figure 3B) was compared with that of previously reported carboxylated multi-walled carbon nanotube (CMWCNT)/CaAlg films [44]. The GO/CaAlg film showed an equilibrium swelling ratio of 239.8%, which was higher than that of CMWCNT/CaAlg films (164.0%). This comparison suggests that GO incorporation affects the swelling behavior of CaAlg hydrogels, while maintaining the structural stability required for subsequent imprinting and sensing applications.

It is worth noting that the water state within alginate hydrogels—ranging from tightly bound water associated with polymer chains and crosslinking junctions to bulk-like free water in the pore spaces—governs protein hydration, mass transport, and the accessibility of imprinted recognition sites. El Hariri El Nokab et al. resolved distinct water pools in alginate hydrogels using solid-state NMR spectroscopy, demonstrating that (re)hydration proceeds through water populations with markedly different mobilities [45]. The observed reduction in swelling is consistent with a more constrained hydrogel network. Changes in water mobility or water–polymer interactions may contribute to this behavior, but the relative fractions of bound and free water were not directly measured.

The tensile strength increased as the GO content increased to 1 wt.% and decreased at higher GO contents. As validated by mechanical testing (Figure 3C), the tensile strength of GO/CaAlg at 1 wt.% loading reached 1921.15 kPa. This significant enhancement is attributed to the two-dimensional structure of GO, whose high aspect ratio provides extensive interfacial contact with the CaAlg matrix and enables effective load distribution. In addition, the oxygen-containing groups on GO may promote interfacial interactions with alginate chains and contribute to stress transfer within the composite network [46,47,48]. Consistent with these mechanisms, GO/CaAlg exhibited the highest fracture energy at 1 wt.% loading (Figure 3D), supporting GO’s role as a multifunctional mechanical reinforcement phase.

### 2.3. Effect of Different Conditions on the Adsorption Performance of GO/CaAlg Films

**Figure 4:**
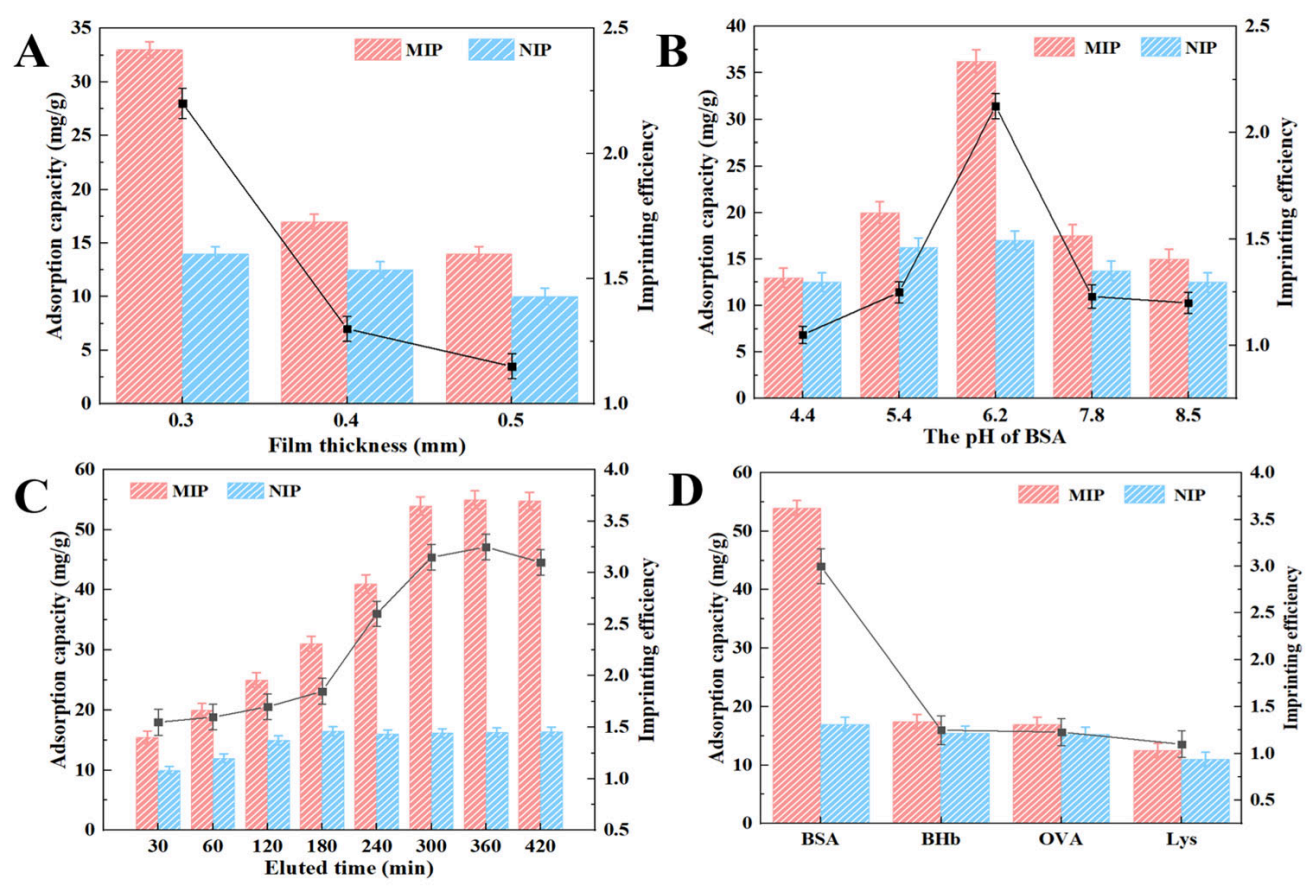
Adsorption capacity and imprinting efficiency of GO/CaAlg MIP and NIP films at different film thicknesses (**A**), protein-solution pH values (**B**), template-elution times followed by BSA rebinding (**C**), and protein types (**D**).

Figure 4A evaluates the adsorption performance of GO/CaAlg MIP and NIP films with varying thicknesses. The results demonstrate that the MIP films exhibit significantly higher adsorption capacity for BSA compared to NIP films. The imprinting efficiency (IE > 2) indicates that the imprinting process markedly enhances the specific binding affinity of the MIP. The film prepared with a nominal casting thickness of 0.3 mm, corresponding to a measured post-cross-linking thickness of 0.107 mm, exhibited the highest BSA adsorption capacity among the thickness conditions tested (Q_e_ = 33.34 mg g^−1^), suggesting that the shorter diffusion pathway facilitates BSA template removal and improves the accessibility of the resulting recognition sites [49]. This thickness-dependent behavior indicates that the accessibility of imprinting sites is strongly governed by the diffusion pathway within the hydrogel interface. Excessively thick films may increase mass-transfer resistance, whereas overly thin films may provide insufficient recognition volume. Further analysis of adsorption behavior across different pH levels revealed that both MIP and NIP films achieved peak adsorption capacity at pH 6.2 (Figure 4B). The selected pH range was designed to cover acidic to mildly alkaline conditions and included pH 4.4, which is close to the isoelectric point of BSA (approximately pH 4.7). At this pH, the net charge of BSA is relatively low, allowing the effect of pH-dependent protein–matrix interactions on BSA rebinding to be evaluated. The additional pH values of 5.4, 6.2, 7.8, and 8.5 were selected to progressively examine the rebinding behavior above the isoelectric point. Under alkaline conditions (pH > 7), changes in the structure and ionization state of BSA and NaAlg may weaken the interactions involved in BSA adsorption [50], diminishing electrostatic interactions and resulting in a substantial decline in adsorption efficiency. Therefore, pH 6.2 was selected as the optimal condition for subsequent BSA adsorption and rebinding experiments.

Figure 4C compares the subsequent BSA rebinding capacities of the MIP and NIP films after treatment with Tris-HCl for different elution times. With increasing elution time, the equilibrium adsorption capacity of the MIP film gradually increased, reaching a maximum value of 55.21 mg g^−1^ and an imprinting efficiency of 3.68 at 360 min. This increase suggests that prolonged elution promoted the progressive release of accessible BSA templates, thereby increasing the availability of recognition sites for subsequent rebinding. Little further change was observed beyond 360 min, indicating that additional elution provided only limited improvement in the rebinding capacity under the experimental conditions. In contrast, the adsorption capacity of the NIP film remained substantially lower and became relatively stable after approximately 120 min of Tris-HCl treatment. Because the NIP film contained no template-derived recognition sites, its adsorption was mainly associated with nonspecific interactions. At 360 min, the adsorption capacity of the NIP film was only 27.2% of that of the MIP film, demonstrating the contribution of the imprinting process to BSA rebinding.

To assess the specificity of the MIP, selectivity experiments were conducted using three reference proteins: bovine hemoglobin (BHb), ovalbumin (OVA), and lysozyme (Lys). These proteins were selected to systematically probe the contributions of molecular size and surface charge to recognition. BHb has a molecular weight (~64.5 kDa) comparable to BSA (~66 kDa) but a different isoelectric point (pI ≈ 6.9 vs. 4.8), allowing evaluation of charge discrimination. OVA has a similar pI (~4.5) to BSA but a smaller molecular weight (~45 kDa), enabling assessment of size selectivity. Lys has a much smaller molecular weight (~14.3 kDa) and a strongly basic pI (~11.0), representing both size and charge dissimilarity. As shown in Figure 4D, the MIP film exhibited the highest equilibrium adsorption capacity for BSA. This enhanced selectivity is attributed to the compatible size and conformation of BSA relative to the imprinted cavities. The lower adsorption capacities for BHb, OVA, and Lys may be attributed to their lower structural and chemical complementarity with the BSA-derived recognition sites, thus confirming the high selectivity of the molecularly imprinted film.

### 2.4. Electrochemical Characterization of Different Modified Electrodes

**Figure 5:**
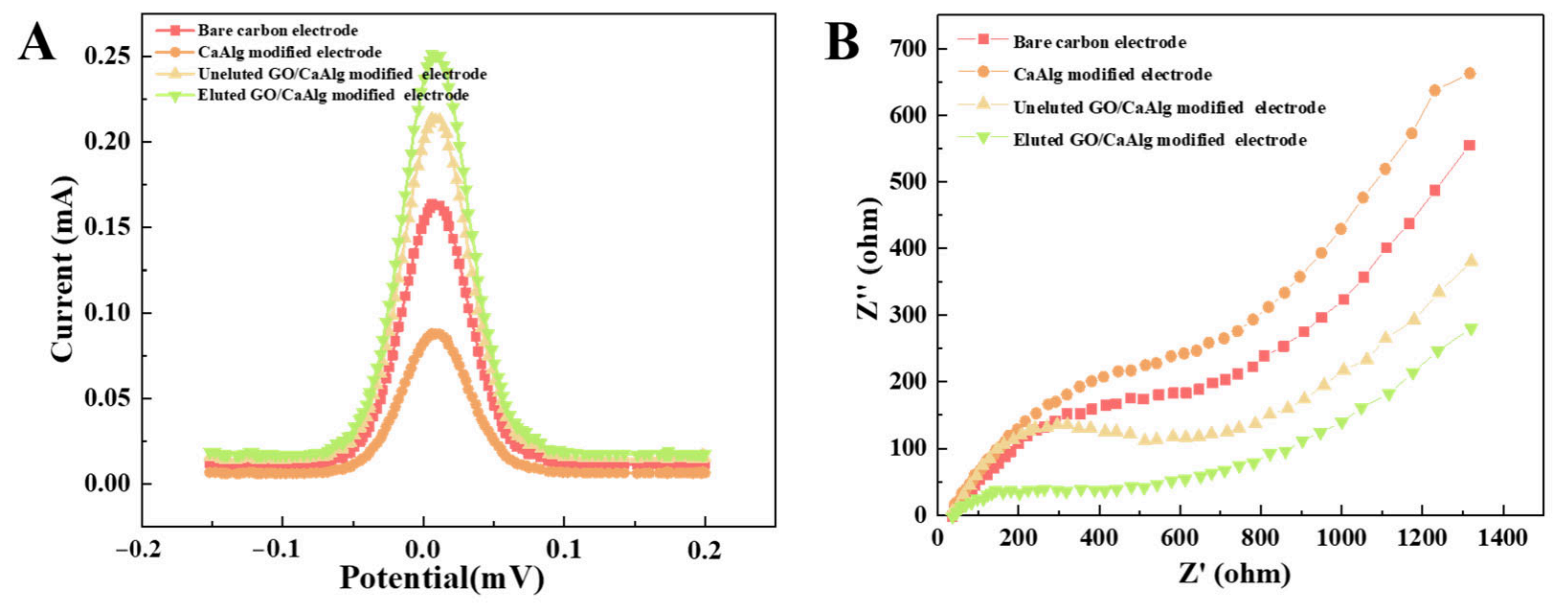
Differential pulse voltammetry (**A**) and electrochemical impedance spectroscopy (**B**) of different modified electrodes.

Electrochemical impedance spectroscopy (EIS) is an effective method for characterizing the electron-transfer kinetics at electrode surfaces [51]. The diameter of the high-frequency semicircle is generally related to the interfacial charge-transfer resistance. As shown in Figure 5A,B, the CaAlg film-modified carbon electrode exhibited a higher interfacial resistance than the bare carbon electrode, which is attributed to the formation of a non-conductive polymer layer that hindered electron transfer [52]. The incorporation of GO altered the interfacial electron-transfer behavior of the hydrogel-modified electrode, resulting in a lower impedance under the investigated conditions [53], thereby accelerating electron transfer. After template elution, the electron-transfer resistance decreased compared with the non-eluted MIP-modified electrode. This can be attributed to the removal of BSA templates, which generated accessible imprinting cavities and diffusion channels, thereby facilitating the transport of [Fe(CN)_6_]^3−/4−^ redox probes toward the electrode surface.

### 2.5. Effect of Different Conditions on GO/CaAlg MIP Film Modified Electrodes

**Figure 6:**
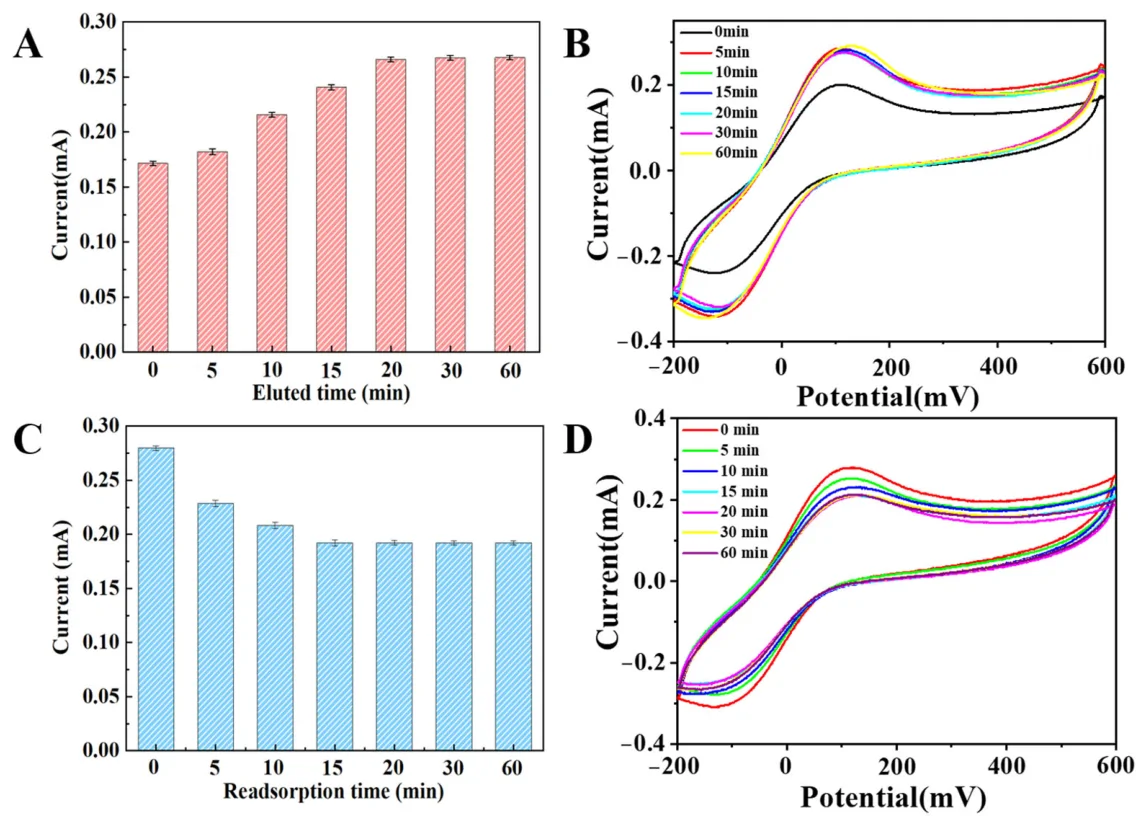
Peak current values (**A**) and cyclic voltammetry curves (**B**) of the MIP-modified electrodes at different elution times; peak current values (**C**) and cyclic voltammetry curves (**D**) of the MIP-modified electrodes at different rebinding times.

To optimize the electrochemical response of the MIP sensor, the elution and rebinding times were investigated. The peak current initially increased with increasing elution time and subsequently reached a plateau (Figure 6A), while the corresponding cyclic voltammetry (CV) curves are shown in Figure 6B. During elution, the interactions between BSA and the imprinted matrix were progressively weakened, promoting the release of BSA from the MIP [54]. The resulting increase in the accessibility of the imprinted sites facilitated the diffusion of the [Fe(CN)_6_]^3−/4−^ redox probe toward the electrode surface, thereby increasing the peak current. When the elution time reached 20 min, the peak current became nearly stable, suggesting that most of the accessible template BSA had been removed under the experimental conditions. The effect of rebinding time on the electrochemical response was subsequently evaluated. At a rebinding time of 0 min, the recognition sites had not yet been occupied by BSA, resulting in a maximum peak current of 0.27 mA (Figure 6C), and the corresponding cyclic voltammetry curves are presented in Figure 6D. As rebinding proceeded, BSA molecules gradually occupied the imprinted sites, hindering the diffusion of the redox probe and electron transfer at the electrode interface. After 15 min, the peak current remained at approximately 0.20 mA, indicating that the rebinding process had approached equilibrium.

**Figure 7:**
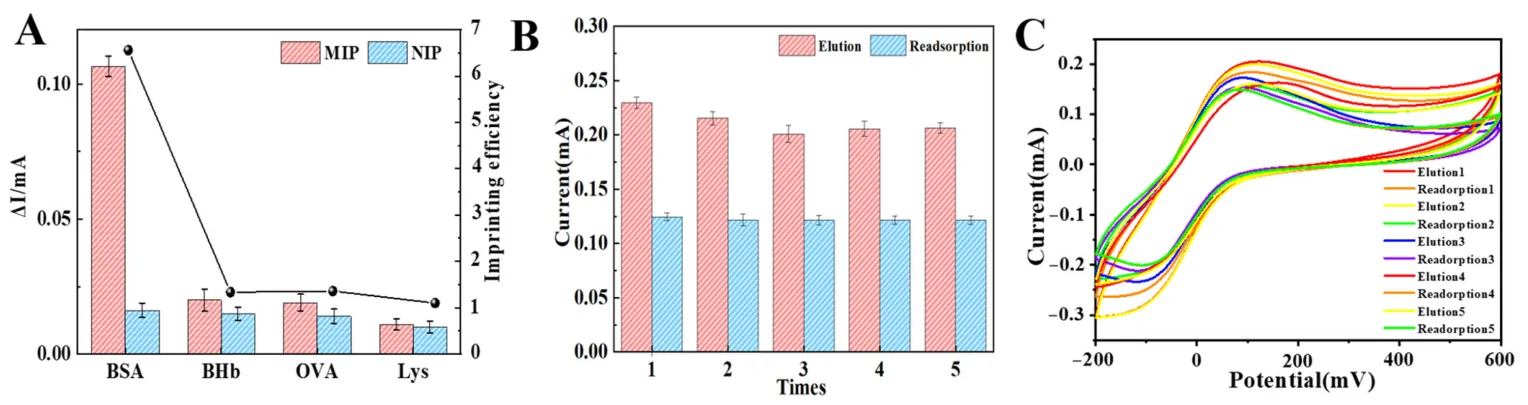
Current changes in the MIP- and NIP-modified electrodes after rebinding different proteins and the corresponding electrochemical imprinting ratios (**A**), peak current values over five template-removal/rebinding cycles (**B**), and corresponding cyclic voltammetry curves (**C**).

To investigate the selective recognition performance of the GO/CaAlg MIP-modified electrode toward BSA, BHb, OVA, and Lys were selected as reference proteins. As shown in Figure 7A, the current change (ΔI) caused by BSA rebinding on the MIP-modified electrode was markedly greater than that on the NIP-modified electrode. This difference can be attributed to the preferential rebinding of BSA to the BSA-derived recognition sites. Occupation of these sites by BSA hindered the transport of the [Fe(CN)_6_]^3−/4−^ redox probe toward the electrode surface, thereby producing a larger current change [55]. The ΔI value for BSA was also considerably higher than those for BHb, OVA, and Lys, which may be associated with the lower structural and chemical complementarity of the reference proteins with the BSA-derived recognition sites. The right *y*-axis in Figure 7A represents the electrochemical imprinting ratio, calculated as ΔI_MIPs_/ΔI_NIPs_ and labeled as imprinting efficiency in the figure. The ratio for BSA was approximately 6.5, which was substantially higher than those for BHb (~1.2), OVA (~1.3), and Lys (~1.1), demonstrating the preferential electrochemical response of the MIP-modified electrode toward BSA [56]. The reusability of the GO/CaAlg MIP-modified electrode was further evaluated over five template-removal/rebinding cycles. As shown in Figure 7B, the peak current after template removal decreased slightly and remained at approximately 0.20 mA, whereas that after BSA rebinding remained relatively stable at approximately 0.12 mA. The corresponding CV curves exhibited similar redox profiles throughout the five cycles (Figure 7C), indicating that the MIP-modified electrode maintained a relatively stable electrochemical response during repeated use.

### 2.6. Antimicrobial Properties of GO/CaAlg MIP Films

**Figure 8:**
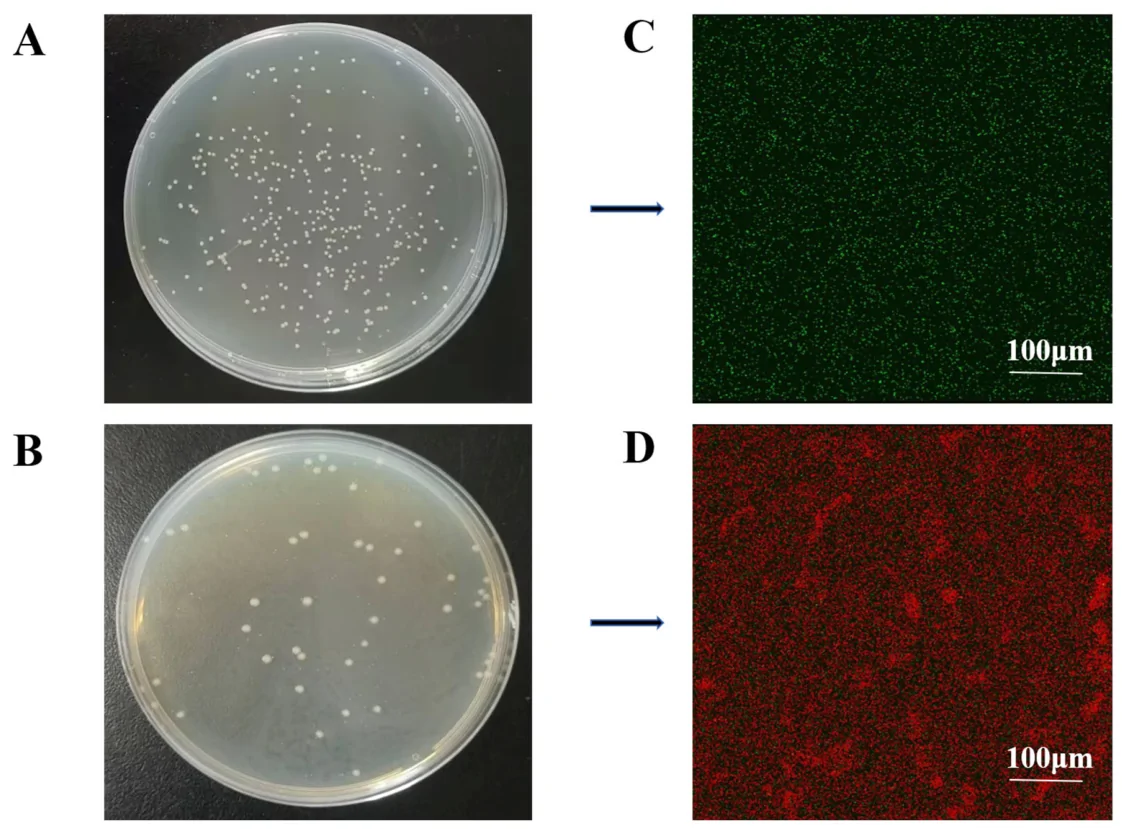
Colony-counting results of the untreated S. aureus control (**A**) and the GO/CaAlg MIP film-treated group (**B**), and corresponding Hoechst 33258/PI fluorescence images of the untreated control (**C**) and film-treated group (**D**).

The antibacterial performance of the GO/CaAlg MIP films against Gram-positive *S. aureus* was evaluated using colony counting and live/dead bacterial staining assays. As shown in Figure 8A,B, the untreated control group yielded 294 ± 8 CFU, whereas the GO/CaAlg MIP film-treated group yielded 46 ± 5 CFU, corresponding to an antibacterial efficiency of 84.35%. Complementary Hoechst 33,258/PI fluorescence staining was performed to examine bacterial membrane integrity. The untreated control predominantly exhibited Hoechst-positive fluorescence with limited PI-positive signals, whereas the GO/CaAlg MIP film-treated group showed markedly stronger PI-positive fluorescence, indicating an increased proportion of bacteria with compromised cell membranes (Figure 8C,D). These qualitative observations were consistent with the colony-counting results, demonstrating the antibacterial activity of the GO/CaAlg MIP film against *S. aureus*.

### 2.7. Comparison with Previously Reported Molecularly Imprinted Materials

To further contextualize the performance of the developed GO/CaAlg MIP film, its key adsorption and molecular recognition properties were compared with those of representative previously reported molecularly imprinted materials. Particular attention was given to BSA binding capacity because it is a direct indicator of the effectiveness of the imprinted recognition sites. The reported adsorbent compositions, template proteins, binding capacities, experimental conditions, and relevant performance parameters are summarized in Table 2.

As shown in Table 2, the present GO/CaAlg MIP film exhibited a BSA binding capacity of 55.21 mg g^−1^ and an imprinting efficiency of 3.68. Compared with previously reported CaAlg-based imprinted hydrogel films, the present material exhibited a higher BSA binding capacity than both the pure CaAlg MIP film (38.6 mg g^−1^) and the CMWCNT/CaAlg MIP film (27.23 mg g^−1^). This difference may be associated with the incorporation of GO, which can modify the physicochemical properties and interfacial characteristics of the CaAlg matrix. It should be noted that some other reported materials, including PGMA/PS MIP microspheres, PEGylated magnetic halloysite nanotube-based MIPs, and poly(ionic liquid)/CaAlg cryogels, exhibited higher BSA binding capacities, ranging from 72.7 to 318.1 mg g^−1^. The differences in binding capacity may be associated with variations in material architecture, surface characteristics, porosity, and experimental conditions. In contrast to these particulate or highly porous materials, the present GO/CaAlg material is a dense, freestanding hydrogel film that can be directly integrated with an electrode interface for electrochemical sensing.

The imprinting efficiency of 3.68 obtained in the present work was higher than those reported for the CMWCNT/CaAlg MIP film (2.73), PEGylated magnetic HNT MIP (2.44), and poly(ionic liquid)/CaAlg cryogel (2.16), but lower than those of the pure CaAlg MIP film (4.2) and PGMA/PS MIP microspheres (4.6). These results indicate that the present GO/CaAlg MIP film provides a considerable BSA binding capacity together with effective formation of selective recognition sites. Overall, the comparison highlights the distinct combination of adsorption, molecular recognition, and hydrogel-film characteristics achieved by the present GO/CaAlg system.

To further clarify the performance characteristics and potential advantages of the developed GO/CaAlg MIP film, its key properties were compared with those of previously reported CaAlg-based BSA molecularly imprinted materials. Two closely related systems, namely CMWCNT/CaAlg and CaAlg/CaSiO_3_, were selected for comparison because they also combine CaAlg-based hydrogel matrices with molecular imprinting for BSA recognition. The comparative results are summarized in Table 3.

The GO/CaAlg film exhibited a tensile strength of 1921.15 kPa, which was higher than those reported for CMWCNT/CaAlg (1440 kPa) and CaAlg/CaSiO_3_ (~1100 kPa), the GO loading in the present study was 1 wt.% relative to the total precursor solution mass, which corresponds to approximately 0.4 wt.% relative to the NaAlg polymer mass. Notably, the BSA adsorption capacity (55.21 mg g^−1^) and imprinting efficiency (3.68) were substantially higher than those reported for CMWCNT/CaAlg (27.23 mg g^−1^ and 2.73, respectively). In addition, the GO/CaAlg MIP film exhibited an antibacterial inhibition rate of 84.35% against *S. aureus*, a functional feature not reported in the previous CaAlg-based imprinted hydrogels.

## 3. Conclusions

GO/CaAlg molecularly imprinted hydrogel films were prepared for selective BSA recognition and integrated with carbon electrodes. GO incorporation reduced swelling and improved the mechanical performance of the CaAlg hydrogel network. At a GO content of 1 wt.%, the tensile strength reached 1921.15 kPa, and the film exhibited the highest fracture energy among the investigated compositions. CD analysis indicated that BSA retained a similar secondary structure before imprinting and after template removal. The MIP film exhibited an equilibrium BSA adsorption capacity of 55.21 mg g^−1^ and an imprinting efficiency of 3.68, together with preferential rebinding of BSA over BHb, OVA, and Lys. At the electrode interface, template removal and BSA rebinding produced opposite changes in the redox-probe current, with the responses reaching plateaus after 20 and 15 min, respectively. The electrochemical imprinting ratio for BSA was approximately 6.5, and the current response remained relatively stable over five template-removal/rebinding cycles. In addition, the GO/CaAlg MIP film exhibited an inhibition rate of 84.35% against *S. aureus*. The simultaneous integration of mechanical reinforcement, selective protein recognition, electrochemical sensing, and antibacterial functionality within a single CaAlg-based imprinted hydrogel highlights the multifunctional characteristics of the present GO/CaAlg platform and demonstrates its potential as a functional interface for selective protein recognition and biomolecular detection.

## 4. Materials and Methods

### 4.1. Materials

Sodium alginate (NaAlg, CAS No. 9005-38-3), ethanol (CAS No. 64-17-5), phosphate-buffered saline (PBS), propidium iodide (PI, CAS No. 25535-16-4), Hoechst 33,258 (CAS No. 23491-45-4), and glutaraldehyde (CAS No. 111-30-8) were purchased from Shanghai Aladdin Bio-Chemical Technology Co., Ltd. (Shanghai, China). Calcium chloride (CaCl_2_, CAS No. 10043-52-4) was obtained from Sinopharm Chemical Reagent Co., Ltd. (Shanghai, China). Sodium chloride (NaCl, CAS No. 7647-14-5) and hydrochloric acid (HCl, CAS No. 7647-01-0) were purchased from Tianjin Chemical Reagent Factory No. 2 (Tianjin, China) and Tianjin Xinghua Chemical Reagent Factory (Tianjin, China), respectively. Tris(hydroxymethyl)aminomethane (Tris, CAS No. 77-86-1) was purchased from the Institute of Biomedical Engineering, Chinese Academy of Medical Sciences (Tianjin, China). Potassium bromide (KBr, CAS No. 7758-02-3) and graphene oxide (GO, CAS No. 1034343-98-0) were obtained from Tianjin United Chemical Reagent Co., Ltd. (Tianjin, China) and Tianjin Kemiou Chemical Reagent Co., Ltd. (Tianjin, China), respectively. Potassium chloride (KCl, CAS No. 7447-40-7), potassium ferricyanide (K_3_[Fe(CN)_6_], CAS No. 13746-66-2), and potassium ferrocyanide trihydrate (K_4_[Fe(CN)_6_]·3H_2_O, CAS No. 14459-95-1) were purchased from Tianjin Fengchuan Chemical Reagent Factory (Tianjin, China). 3-Aminopropyltriethoxysilane (KH550, CAS No. 919-30-2) was purchased from Tianjin Heowns Biochemical Technology Co., Ltd. (Tianjin, China). Bovine serum albumin (BSA), bovine hemoglobin (BHb), ovalbumin (OVA), and lysozyme (Lys) were purchased from Shanghai Langji Technology Development Co., Ltd. (Shanghai, China). Bacterial nutrient medium and agar were obtained from Shanghai Aoboxing Biotechnology Co., Ltd. (Shanghai, China). Screen-printed carbon electrodes, hereafter referred to as carbon electrodes, were provided by the Advanced Materials and Printed Electronics Center, Tianjin University of Technology (Tianjin, China). Data analysis and graph plotting were performed using Origin software (Version 2024, OriginLab Corporation, Northampton, MA, USA). Reference management was performed using EndNote software (Version 21.4, Clarivate Analytics, Philadelphia, PA, USA).

### 4.2. Preparation of BSA-Imprinted GO/CaAlg Film

For the GO-containing samples, predetermined amounts of GO were dispersed in deionized water and ultrasonicated for 2 h. The GO content, expressed as wt.% relative to the total mass of NaAlg, was varied from 0 to 3 wt.%. The GO-free sample was prepared using the same procedure without the addition of GO. Subsequently, BSA was added to each dispersion to a final concentration of 0.13 wt.% and stirred until completely dissolved. NaAlg was then slowly added to a final concentration of 2.5 wt.% under continuous stirring until a homogeneous precursor solution was obtained. Approximately 5 mL of the resulting precursor solution was poured onto a clean, smooth glass plate (10 cm × 10 cm). The solution was uniformly spread using a glass rod with copper wires of predetermined diameters wrapped around both ends to control the casting gap. The preparation variables and their corresponding levels are summarized in Table 4. The glass plate was then immersed in a 2.5 wt.% CaCl_2_ solution for 8 h to achieve ionic cross-linking, yielding a BSA-containing GO/CaAlg hydrogel film [62]. The template BSA was removed by treating the resulting film with Tris-HCl buffer (pH 7.4) for 360 min. After elution, the film was thoroughly rinsed with deionized water to obtain the GO/CaAlg MIP film. The corresponding NIP film was prepared using the same procedure without the addition of BSA.

The film thickness was controlled by pouring a fixed volume of the GO/NaAlg solution onto a glass plate and spreading it with a glass rod wrapped with copper wires of different diameters (0.3, 0.4, or 0.5 mm), which were used to define the film thickness during casting. After Ca^2+^ cross-linking, the actual film thickness was measured using a digital thickness gauge. Five measurements were taken at different positions for each film, and the maximum and minimum values were excluded; the remaining three measurements were averaged. The resulting average film thicknesses after cross-linking were 0.107, 0.185, and 0.239 mm for films prepared with nominal thicknesses of 0.3, 0.4, and 0.5 mm, respectively.

### 4.3. Preparation of GO/CaAlg MIP-Modified Electrodes

**Figure 9:**
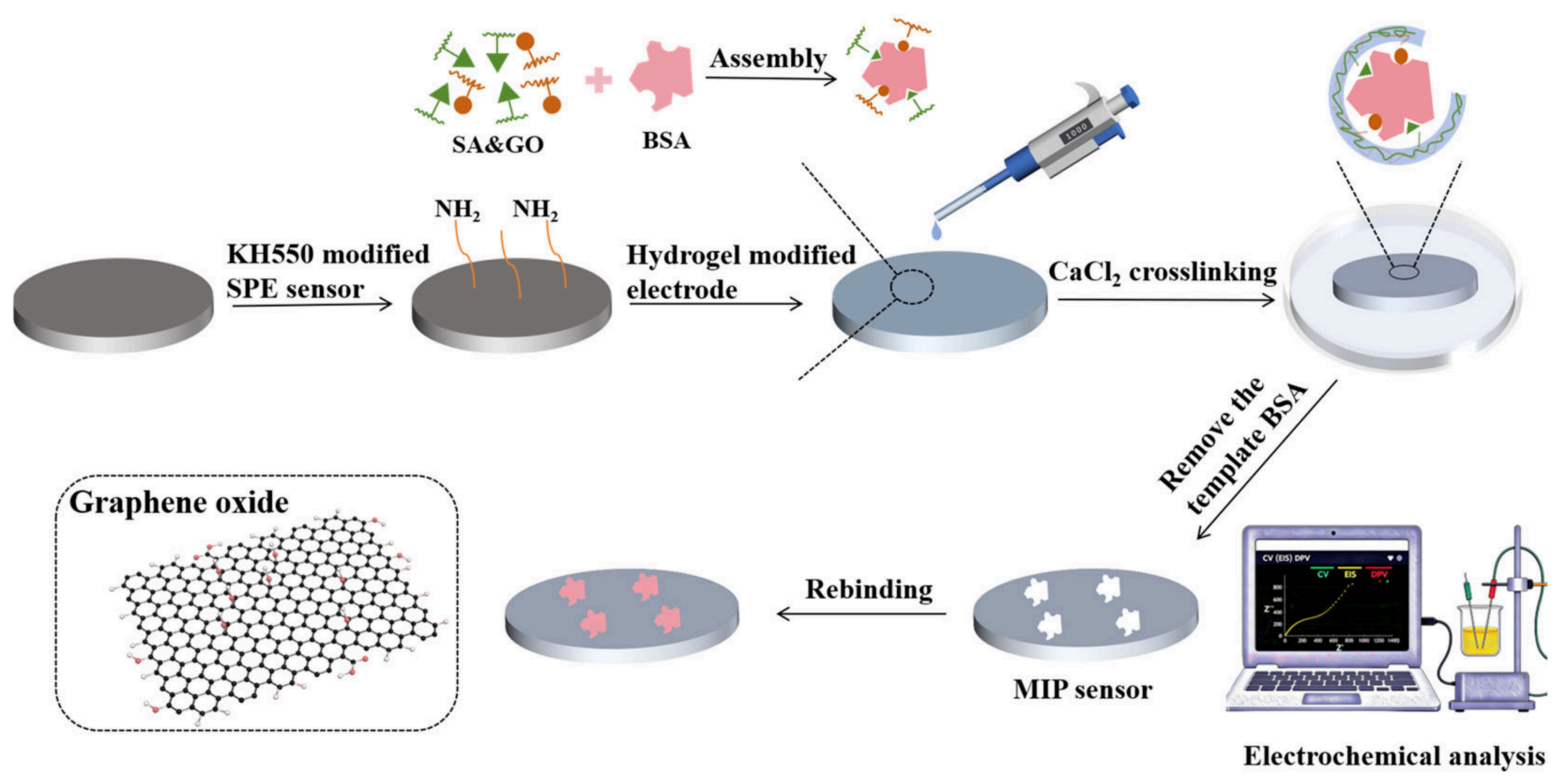
Fabrication process of the GO/CaAlg MIP-modified electrode.

Figure 9 illustrates the fabrication process of the GO/CaAlg MIP-modified electrode. First, a bare carbon electrode was immersed in ethanol for 1 h to remove surface contaminants and subsequently modified with KH550 for 1 h. The BSA-containing GO/CaAlg precursor solution was then uniformly coated onto the surface of the KH550-modified carbon electrode. The coated electrode was immersed in a 2.5 wt.% CaCl_2_ solution for 1 h to achieve ionic cross-linking. The template BSA was subsequently removed using Tris-HCl buffer (pH 7.4) for 20 min, followed by thorough rinsing with deionized water, to obtain the GO/CaAlg MIP-modified electrode. The corresponding NIP-modified electrode was prepared using the same procedure with a precursor solution containing no BSA.

### 4.4. Characterizations

The surface morphology of GO/CaAlg hydrogel imprinted films before and after elution was observed using scanning electron microscopy (SEM; S-4800, Hitachi, Tokyo, Japan) at an accelerating voltage of 10 kV. The elemental composition of the film surface was analyzed using an energy-dispersive X-ray spectroscopy system (EDS; Apollo XL, EDAX, Mahwah, NJ, USA) coupled with the SEM instrument. The analyzed area corresponded to the surface of the hydrogel film. The samples were analyzed using Fourier transform infrared (FTIR) spectroscopy (VECTOR-22, Bruker, Ettlingen, Germany). The conformational changes in BSA solutions before and after elution (Tris-HCl eluent, pH 7.4) were detected using circular dichroism (CD) spectroscopy (MOS-450/AF-CD, BioLogic, Grenoble, France). All experiments were performed in triplicate (*n* = 3), and the results are presented as the mean ± standard deviation (SD).

### 4.5. Swelling Test of GO/CaAlg Films

GO/CaAlg films with different GO contents were cut into circular specimens of the same area using a punch. After the surface solution was gently removed with filter paper, the initial mass of each specimen was recorded. The specimens were then immersed in 10 mL of 0.9 wt.% NaCl solution. At predetermined intervals, the specimens were removed, gently blotted with filter paper, and weighed. This procedure was repeated until the specimen mass remained constant or the film ruptured. The swelling rate Rs (%) was calculated as follows:

(1) $${\mathrm{Rs}\;=\;(W}_{t}\;-{\;W}_{0}{)/W}_{0}\;\times \;100\%$$

where W_0_ is the initial mass and W_t_ is the mass at different points in time.

### 4.6. Tensile Testing of GO/CaAlg Films

GO/CaAlg hydrogel films with different GO contents were cut into rectangular specimens measuring 5 mm in width and 20 mm in length. The mechanical properties of the films were measured using an LLY-06F tensile testing machine (Laizhou Electronic Instrument Co., Ltd., Yantai, China). Before testing, the instrument parameters were set as follows: temperature, 25 °C; crosshead speed, 10 mm min^−1^; fracture threshold, 80%; initial gauge length, 10 mm; and linear density, 1 dTex.

(2) $$\lambda =(L-L_{0}{)/L}_{0}\;\times \;100\%$$

where L_0_ is the initial gauge length and L is the specimen length at fracture.

### 4.7. Protein Adsorption on GO/CaAlg MIP Films

The protein adsorption performance of the GO/CaAlg MIP films was evaluated using the corresponding NIP films as controls. The effects of film thickness (0.3, 0.4, and 0.5 mm), protein-solution pH (4.4, 5.4, 6.2, 7.8, and 8.5), and template-elution time (30–420 min) on BSA rebinding were systematically investigated. For the elution-time experiment, separate MIP films were treated with Tris-HCl solution for predetermined periods, while the corresponding NIP films underwent the same treatment as controls. After thorough rinsing with deionized water, the films were incubated in BSA solutions under identical adsorption conditions until equilibrium was reached. Molecular selectivity was evaluated using BSA as the template protein and BHb, OVA, and Lys as reference proteins. The MIP and NIP films were separately incubated with each protein solution at the same initial concentration under identical adsorption conditions.

After adsorption equilibrium was reached, the residual protein concentration in the solution was determined using a UV–vis spectrophotometer by recording the absorbance at 278 nm. The calibration equation for BSA determination was A = 1.1723 C − 0.00551 (R^2^ = 0.99875) over a linear range of 0.071–0.85 mg mL^−1^, with a limit of detection (LOD) of 0.021 mg mL^−1^ and a limit of quantification (LOQ) of 0.071 mg mL^−1^ (Text S3). The equilibrium adsorption capacity, Q_e_ (mg g^−1^), was calculated as follows:

(3) $$Q_{e}{\;=\;(C}_{0}\;-{\;C}_{e})\;\times \;V/W$$

where C_0_ and C_e_ are the initial and equilibrium protein concentrations (mg mL^−1^), respectively; V is the volume of the protein solution (mL); and W is the mass of the MIP or NIP film (g).

The imprinting efficiency, IE, was determined according to:

(4) $${\mathrm{IE}\;=\;Q}_{\mathrm{MIP}}{/Q}_{\mathrm{NIP}}$$

where Q_MIP_ and Q_NIP_ are the equilibrium adsorption capacities of the MIP and NIP films for the corresponding protein, respectively. Detailed experimental procedures are provided in Supporting Information Text S1.

### 4.8. Electrochemical Testing of GO/CaAlg MIP Sensors

The MIP- and NIP-modified electrodes were used as working electrodes, with platinum sheets and bare carbon electrodes serving as auxiliary and reference electrodes, respectively, for electrochemical measurements. The buffer solution was prepared by dissolving 0.0845 g K_4_[Fe(CN)_6_]·3H_2_O, 0.0659 g K_3_[Fe(CN)_6_], 3.152 g Tris, and 5.9641 g KCl in 200 mL distilled water. The [Fe(CN)_6_]^3−^/[Fe(CN)_6_]^4−^ redox couple was used as the electrochemical probe to monitor the current changes in the BSA molecularly imprinted sensor. Electrochemical measurements were performed at a scanning rate of 50 mV·s^−1^.

### 4.9. Antibacterial Test of GO/CaAlg MIP Films

The antibacterial activity of the GO/CaAlg MIP films against *Staphylococcus aureus* was evaluated using colony-counting and fluorescence-staining assays. Before the antibacterial tests, the film samples were sterilized by ultraviolet irradiation for 30 min on each side.

For colony counting, a single bacterial colony was inoculated into 10 mL of liquid nutrient medium and cultured with shaking at 37 °C for 12 h. The bacterial suspension was subsequently diluted to 1 × 10^6^ colony-forming units (CFU) mL^−1^. Each sterilized film sample was immersed in 3 mL of the bacterial suspension and incubated at 37 °C for 24 h. After incubation, the bacterial suspension was serially diluted to 10^−6^, spread onto agar plates, and cultured at 37 °C for another 24 h before colony counting. The antibacterial efficiency was calculated as follows:

(5) $${\mathrm{Antibacterial}\;\mathrm{efficiency}\;(\%)\;=\;(N}_{0}-N_{1}{)/N}_{0}\;\times \;100$$

where N_0_ and N_1_ are the colony numbers of the blank control and film-treated groups, respectively. Fluorescence staining was further performed to visualize bacterial viability after contact with the hydrogel films. Detailed staining and imaging procedures are provided in Supporting Information Text S2.

## Figures and Tables

**Table 1:** Surface element content of GO/CaAlg MIP hydrogel film after removing BSA.

Hydrogel Film	Element	Content (wt.%)
GO/CaAlg MIP (after BSA removal)	C	50.61
	O	40.93
	Ca	4.66
	N	1.02
	Na	2.78

**Table 2:** Comparison of BSA binding capacities and relevant performance parameters of previously reported molecularly imprinted materials.

Material	BSA Binding Capacity, Q_e_ (mg g^−1^)	pH	Adsorption Time	Detection Method	IE	Ref.
GO/CaAlg MIP	55.21	6.2	360 min	UV–vis	3.68	This work
Pure CaAlg MIP film	38.6	6.2	240 min	UV–vis	4.2	[57]
CMWCNT/CaAlg MIP film	27.23	7.4	300 min	UV–vis	2.73	[44]
PGMA/PS MIP microspheres	72.7	No report	No report	UV–vis	4.6	[58]
PEGylated magnetic HNT MIP	258.4	7.0	80 min	UV–vis	2.44	[59]
Poly(ionic liquid)/CaAlg cryogel	318.1	7.4	360 min	UV–vis	2.16	[60]

**Table 3:** Comparison of the key properties of the GO/CaAlg MIP film with previously reported CaAlg-based BSA-imprinted systems.

Property	GO/CaAlg	CMWCNT/CaAlg [44]	CaAlg/CaSiO_3_ [61]
Reinforcing filler (loading)	GO (1 wt.%)	CMWCNT (2 wt.%)	Na_2_SiO_3_ (25 wt.%)
Tensile strength	1921 kPa	1440 kPa	~1100 kPa
BSA adsorption capacity	55.21 mg g^−1^	27.23 mg g^−1^	No report
Imprinting efficiency	3.68	2.73	No report
Antibacterial activity	84.35% (*S. aureus*)	No report	No report

**Table 4:** Preparation variables and their corresponding levels for GO/CaAlg hydrogel films.

Preparation Variable	Levels
GO content (wt.%)	0, 0.5, 1, 2, 3
Copper-wire diameter (mm)	0.3, 0.4, 0.5

## Data Availability

Data are contained within the article and Supplementary Materials.

## Supplementary Materials

The following supporting information can be downloaded at: https://www.mdpi.com/article/10.3390/gels12100913/s1,Text S1: Detailed procedures for protein adsorption and molecular selectivity; Text S2: Detailed procedures for antibacterial evaluation; Text S3: Calibration curve for BSA determination by UV–Vis spectroscopy; Figure S1: Calibration curve for BSA determination by UV–Vis spectroscopy at 278 nm.

## Abbreviations

BHb	Bovine hemoglobin
BSA	Bovine serum albumin
CaAlg	Calcium alginate
CD	Circular dichroism
CFU	Colony-forming units
CLSM	Confocal laser scanning microscopy
CV	Cyclic voltammetry
EIS	Electrochemical impedance spectroscopy
FTIR	Fourier transform infrared spectroscopy
GO	Graphene oxide
IE	Imprinting efficiency
KH550	3-Aminopropyltriethoxysilane
Lys	Lysozyme
MIP	Molecularly imprinted polymer
MIT	Molecular imprinting technology
NaAlg	Sodium alginate
NIP	Non-imprinted polymer
OVA	Ovalbumin
PBS	Phosphate-buffered saline
PI	Propidium iodide
Q_e_	Equilibrium adsorption capacity
Rs	Swelling rate
SEM	Scanning electron microscope
Tris	Tris(hydroxymethyl)aminomethane
