# Supplementary material for: Graphene Oxide-Regulated Calcium Alginate Molecularly Imprinted Hydrogel Films with Enhanced Physico-Mechanical Properties and Selective Protein Recognition
Source: Gels. 2026 Oct 10;12(10):913. doi: 10.3390/gels12100913 (PMC13704315; doi:10.3390/gels12100913)
Supplement: Supplementary file 1 [file gels-12-00913-s001.zip › gels-4584809-supplementary.pdf]

Supplementary Material

Graphene oxide-regulated calcium alginate molecularly imprinted hydrogel films with enhanced physico-mechanical properties and selective protein recognition

Siyu Wang [1], Haojie Li [1], Ying Zhang[1], Zhengchun Yang [2]* and Kongyin Zhao*[1]

[1] State Key Laboratory of Advanced Separation Membrane Materials, School of Material Science and engineering, Tiangong University, Tianjin 300387, China; 2431020315@tiangong.edu.cn (S.W.); 2531020359@tiangong.edu.cn (H.L.); 2530020266@tiangong.edu.cn (Y.Z.); zhaokongyin@tiangong.edu.cn (K.Z.)

[2] School of Integrated Circuit Science and Engineering, Advanced Materials and Printed Electronics Center, Tianjin University of Technology, Tianjin 300384, China. eieyz@tjut.edu.cn (Z.Y.)

* Correspondence: zhaokongyin@tiangong.edu.cn (K.Z.); Tel./Fax: +86-022-83955055. eieyz@tjut.edu.cn (Z.Y.), Tel./Fax: +86-022-60210570.

List of Supplementary Material:

Text S1. Detailed procedures for protein adsorption and molecular selectivity.

Text S2. Detailed procedures for antibacterial evaluation.

Text S3. Calibration curve for BSA determination by UV–Vis spectroscopy.

Text S1. Detailed procedures for protein adsorption and molecular selectivity

The protein adsorption performance of the GO/CaAlg molecularly imprinted polymer (MIP) films was investigated using the corresponding non-imprinted polymer (NIP) films as controls. The effects of film thickness, protein-solution pH, template-elution time, and protein type on the adsorption and recognition behavior of the hydrogel films were systematically evaluated.

To investigate the effect of film thickness, MIP and NIP films with thicknesses of 0.3, 0.4, and 0.5 mm were used for bovine serum albumin (BSA) adsorption. The influence of solution pH was evaluated using BSA solutions with pH values of 4.4, 5.4, 6.2, 7.8, and 8.5. To optimize the template-elution process, the MIP films were eluted for predetermined periods ranging from 30 to 420 min and subsequently subjected to BSA rebinding under identical adsorption conditions.

The molecular selectivity of the imprinted films was evaluated using BSA as the template protein and bovine hemoglobin (BHb), ovalbumin (OVA), and lysozyme (Lys) as reference proteins. The MIP and NIP films were separately immersed in the corresponding protein solutions under the same experimental conditions. After adsorption equilibrium was reached, the residual protein concentration in the solution was determined using a UV-Vis spectrophotometer. The absorbance values at 278 and 330 nm were recorded. The equilibrium adsorption capacity, Q_e, and the imprinting efficiency, *IE,* were calculated using the equations provided in Section 4.7 of the main text.

Text S2. Detailed procedures for antibacterial evaluation

The antibacterial activity of the GO/CaAlg MIP films against Staphylococcus aureus was evaluated using fluorescence staining and colony-counting assays.

Hoechst 33258/PI live/dead bacterial staining assay

Circular GO/CaAlg MIP film samples with a diameter of 2 cm were sterilized and placed in 12-well plates. Subsequently, 3 mL of an *S. aureus* suspension at a concentration of approximately 1×10^8 CFU mL^{-1} was added to each well, followed by incubation at 37 °C for 24 h. After incubation, the bacterial suspensions were collected and centrifuged at 3000 rpm for 5 min. The supernatants were discarded, and the bacterial cells were washed twice with phosphate-buffered saline (PBS). The bacterial cells were then stained with Hoechst 33258 and propidium iodide (PI) for 30 min at room temperature in the dark. After staining, the cells were fixed with 2.5% glutaraldehyde for 4 h and dehydrated using a graded ethanol series. The treated bacterial suspensions were deposited onto glass slides, air-dried, and observed using confocal laser scanning microscopy (CLSM; TCS SP8, Leica, Germany). Hoechst 33258 fluorescence was used to visualize the total bacterial population, whereas PI fluorescence indicated bacteria with compromised cell membranes.

Colony-counting assay

Before testing, the GO/CaAlg MIP film samples were sterilized by ultraviolet irradiation for 30 min on each side. A single bacterial colony was inoculated into 10 mL of liquid nutrient medium and cultured with shaking at 37 °C for 12 h. The resulting bacterial suspension was subsequently diluted with liquid nutrient medium to the required concentration.

Each sterilized film sample was immersed in 3 mL of bacterial suspension in a 12-well plate and incubated at 37 °C for 24 h. After incubation, the recovered bacterial suspension was serially diluted to 10^{-6}, spread onto agar plates, and cultured at 37 °C for another 24 h. The bacterial colonies were then counted, and the antibacterial efficiency was calculated using the equation provided in Section 4.9 of the main text.

Text S3. Calibration curve for BSA determination by UV–Vis spectroscopy

A standard curve was established by ultraviolet–visible (UV–Vis) spectrophotometry to determine the relationship between the concentration and absorbance of bovine serum albumin (BSA). The resulting regression equation was subsequently used to quantify the BSA concentration in the adsorption experiments.

(1) Preparation of BSA standard solutions

An appropriate amount of BSA standard was accurately weighed and dissolved in deionized water (pH adjusted to 6.2) to prepare a stock solution. The stock solution was then serially diluted to obtain standard solutions with concentrations of 0, 0.03, 0.08, 0.25, 0.43, and 0.85 mg·mL^{-1}.

(2) Absorbance measurement

The absorbance of each standard solution was measured at 278 nm using a UV–Vis spectrophotometer at room temperature, corresponding to the characteristic absorption wavelength of BSA. Each concentration was measured in triplicate, and the average absorbance value was used for subsequent analysis.

(3) Construction of the standard curve

The standard curve was constructed by plotting the absorbance at 278 nm (A) against the BSA concentration (C, mg·mL^{-1}). Linear regression analysis was performed to obtain the regression equation and the coefficient of determination (R^2). The standard curve was fitted according to Equation (S1):

$$A=kc+b \quad \text{(S1)}$$

where A is the absorbance at 278 nm, c is the BSA concentration (mg·mL^{-1}), k is the slope of the regression line, and b is the intercept.

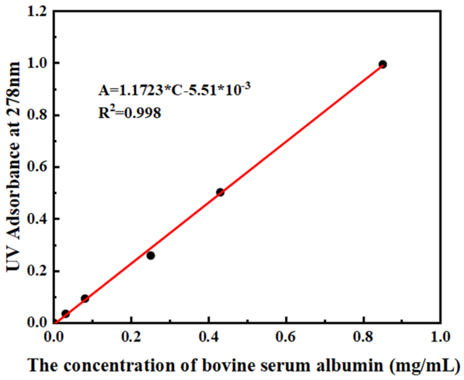

Figure S1. Calibration curve for BSA determination by UV–Vis spectroscopy at 278 nm.
